# Increased Soluble PD-1 Predicts Response to Nivolumab plus Ipilimumab in Melanoma

**DOI:** 10.3390/cancers14143342

**Published:** 2022-07-09

**Authors:** Jesper Geert Pedersen, Mateo Sokac, Boe Sandahl Sørensen, Adam Andrzej Luczak, Ninna Aggerholm-Pedersen, Nicolai Juul Birkbak, Trine Heide Øllegaard, Martin Roelsgaard Jakobsen

**Affiliations:** 1 Department of Biomedicine, Aarhus University, 8000 Aarhus C, Denmark; jgp@biomed.au.dk; 2 Department of Molecular Medicine (MOMA), Aarhus University Hospital, 8200 Aarhus N, Denmark; mateo.sokac@clin.au.dk (M.S.); nbirkbak@clin.au.dk (N.J.B.); 3 Department of Clinical Biochemistry, Aarhus University Hospital, 8200 Aarhus N, Denmark; boesoere@rm.dk; 4 Department of Oncology, Aalborg University Hospital, 9000 Aalborg, Denmark; adal@rn.dk; 5 Department of Oncology, Aarhus University Hospital, 8200 Aarhus N, Denmark; aggerholm@oncology.au.dk; 6 Department of Clinical Medicine, Aarhus University, 8200 Aarhus N, Denmark; 7 Bioinformatics Research Centre, Aarhus University, 8000 Aarhus C, Denmark

**Keywords:** immunotherapy, melanoma, liquid biopsies, inflammatory cytokines, PD-1

## Abstract

**Simple Summary:**

Checkpoint inhibitors have emerged as an effective therapy for patients with metastatic melanoma significantly improving survival for these patients. Despite this, many patients do not respond to the therapy and no current biomarkers can identify responders from non-responders. Using machine learning, we analysed cytokine levels in serially collected liquid biopsy to identify cytokine changes associated with response to checkpoint inhibitors in advanced-stage melanoma patients. The results presented here highlight that serial measurements of cytokine levels are a strong predictor of treatment response. Particularly, we demonstrate that high increases of soluble PD-1 measured from baseline to on-treatment is significantly associated with superior PFS in patients treated with nivolumab plus ipililumab. These results suggest that monitoring cytokine levels using serial samples is informative of treatment response and can improve guidance of treatment modality and the outcome of cancer patients.

**Abstract:**

Background: Checkpoint inhibitors have revolutionized the treatment of metastatic melanoma, yielding long-term survival in a considerable proportion of the patients. Yet, 40–60% of patients do not achieve a long-term benefit from such therapy, emphasizing the urgent need to identify biomarkers that can predict response to immunotherapy and guide patients for the best possible treatment. Here, we exploited an unsupervised machine learning approach to identify potential inflammatory cytokine signatures from liquid biopsies, which could predict response to immunotherapy in melanoma. Methods: We studied a cohort of 77 patients diagnosed with unresectable advanced-stage melanoma undergoing treatment with first-line nivolumab plus ipilimumab or pembrolizumab. Baseline and on-treatment plasma samples were tested for levels of PD-1, PD-L1, IFNγ, IFNβ, CCL20, CXCL5, CXCL10, IL6, IL8, IL10, MCP1, and TNFα and analyzed by Uniform Manifold Approximation and Projection (UMAP) dimension reduction method and k-means clustering analysis. Results: Interestingly, using UMAP analysis, we found that treatment-induced cytokine changes measured as a ratio between baseline and on-treatment samples correlated significantly to progression-free survival (PFS). For patients treated with nivolumab plus ipilimumab we identified a group of patients with superior PFS that were characterized by significantly higher baseline-to-on-treatment increments of PD-1, PD-L1, IFNγ, IL10, CXCL10, and TNFα compared to patients with worse PFS. Particularly, a high PD-1 increment was a strong individual predictor for superior PFS (HR = 0.13; 95% CI 0.034–0.49; *p* = 0.0026). In contrast, decreasing levels of IFNγ and IL6 and increasing levels of CXCL5 were associated with superior PFS in the pembrolizumab group, although none of the cytokines were individually predictors for PFS. Conclusions: In short, our study demonstrates that a high increment of PD-1 is associated with superior PFS in advanced-stage melanoma patients treated with nivolumab plus ipilimumab. In contrast, decreasing levels of IFNγ and IL6, and increasing levels of CXCL5 are associated with response to pembrolizumab. These results suggest that using serial samples to monitor changes in cytokine levels early during treatment is informative for treatment response.

## 1. Background

Historically, metastatic melanoma (MM) has been associated with poor prognosis and limited treatment options. However, the introduction of various immunotherapies during the last decade has revolutionized treatment for MM patients. Particularly, checkpoint inhibitors targeting programmed cell death protein 1 (PD-1) as monotherapy or in combination with cytotoxic T-lymphocyte-associated protein 4 (CTLA-4) inhibitors have significantly improved survival for a sub-group of MM patients [1,2]. Nevertheless, for a considerable part of the patients we still observe a lack of therapy response. Thus, as many as 42% of the patients treated with the combination of anti-PD-1 and anti-CTLA-4 inhibitors do not respond to the therapy [3]. For the anti-PD-1 mono-therapy non-responders make up 54% of the patients [4].

In general, anti-tumor immune activation is believed by many to be crucial for mounting a proper therapy response. This includes antigen presentation and T cell activation, trafficking and infiltration of activated T cells into the tumor, and tumor-killing activity by T cells within the tumor microenvironment [5]. Such hypothesis is supported by the observations that high tumor density of CD8 T cells [6,7], high Interferon-γ (IFNγ)-related gene expression signature within the tumor [8], and high tumor mutational burden [9,10,11] all are associated with response to checkpoint inhibitors in melanoma patients. Furthermore, as checkpoint inhibitors reinvigorate the immune system, evaluating the treatment-induced immune responses during treatment could be a specific measure of treatment efficacy. However, given the difficulties of sampling and measuring intra-tumoral biomarkers, much focus has recently moved to identifying immune-related biomarkers in liquid biopsies.

Liquid biopsies have the advantage of being minimally invasive and easy to obtain, allowing for frequent sampling. However, when and how to sample may affect response predictions based on liquid biopsy-based biomarkers. Some studies have shown that high pre-treatment plasma levels of IFNγ, Interleukin 6 (IL6), and IL10 [12] and Transforming Growth Factor β (TGF-β) [13] are associated with response to anti-PD-1 therapy in melanoma patients. In contrast, high pre-treatment levels of IL8 are associated with poor overall survival (OS) in a large cohort of melanoma patients [14]. Adding to this, another study demonstrated that decreasing IL8 serum levels upon treatment were highly specific for response to anti-PD1 therapy [15]. Finally, other studies find that high pre-treatment levels of PD-1 and Programmed death ligand 1 (PD-L1) are associated with disease progression [16,17,18]. However, whether systemic cytokine levels, in general, are affected by checkpoint inhibitors and how this correlates to treatment response remains elusive.

In this study, we applied an unbiased machine learning workflow including Uniform Manifold Approximation and Projection (UMAP) and cluster analysis to evaluate the potential of a plasma-based cytokine profile to predict response to checkpoint inhibitor therapy in advanced-stage MM patients. By analyzing cytokine changes as ratios from baseline to on-treatment samples, we identified a group of patients with superior Progression-free survival (PFS). Based on this, we identified soluble PD-1 to be a strong predictor for PFS in the nivolumab plus ipilimumab treatment group, whereas IFNγ, IL6 and CXCL5 predicted response to pembrolizumab.

## 2. Material and Methods

### 2.1. Patients and Treatment

Patients with previously untreated, unresectable stage III or stage IV metastatic melanoma who received systemic treatment with immune checkpoint inhibitors were eligible for the study. Key inclusion criteria were the absence of uveal melanoma, absence of another primary cancer, and no previous diagnosis with cancer. Patients were enrolled between August 2017 and July 2019. All consecutive patients referred to systemic treatment at the Department of Oncology, Aarhus University Hospital (Denmark) or Aalborg University Hospital (Denmark) were included in the study. All patients gave informed written consent before inclusion. The study was approved by the Central Denmark Region Committees on Biomedical Research Ethics (no. 1-10-72-374-15) and performed in accordance with the Declaration of Helsinki.

Patients received pembrolizumab at a dose of 2 mg/kg every three weeks or nivolumab 1 mg/kg plus ipilimumab 3 mg/kg every three weeks, followed by maintenance nivolumab 1 mg/kg.

Thirty-six healthy age- and gender-matched control donors were recruited from Aarhus University Hospital Blood Bank. A total of 15 female and 21 male healthy donor controls were collected with an age range of 52–68 years (mean age 62.3 years).

### 2.2. Disease Characteristics and Response Assessment

Patient demographics and clinicopathologic features included: Eastern Cooperative Oncology Group (ECOG) performance status, lactate dehydrogenase (LDH) levels, C-reactive protein (CRP) levels, and any adverse events requiring steroid treatment during the first year of treatment. Elevated LDH level was defined as above 205 units/liter (U/L) for patients below or at the age of 70, and above 255 U/L for patients above the age of 70. Tumour biopsies were routinely screened for BRAF^V600E^ mutational status and PD-L1 expression (</>1%).

Treatment responses were evaluated by Positron emission tomography/computed tomography (PET/CT) scans and/or CT of chest, abdomen, and pelvis, and magnetic resonance imaging (MRI) in case of known brain metastases.

### 2.3. Sample Collection and Processing

Peripheral blood samples (10 mL, Ethylenediaminetetraacetic acid (EDTA) tubes, BD Vacutainer, Plymouth, UK) were obtained at baseline before treatment initiation (median 0 days, range 0–30 days) and on-treatment (median 62 days post-treatment initiation, range 32–91 days). Plasma was isolated by centrifugation at 950× *g* for 25 min and cryopreserved at −80 °C until analysis.

### 2.4. Plasma Cytokine Analysis

Plasma levels of IFNβ, IFNγ, IL6, IL8, IL10, CXCL10, CXCL5, CCL20, TNFα, MCP1, PD-1, and PD-L1 were quantified by commercially available Meso Scale kits (Meso Scale Discovery, Rockville, MD, USA, cat# K15067, F214A-3 and F214C-3) in cryopreserved plasma samples from baseline and on-treatment samples according to manufacturer’s protocol. Data was acquired using a QuickPlex SQ 120 instrument (Meso Scale Discovery, Rockville, MD, USA). Samples below lower limit of quantification were set to zero.

### 2.5. Statistical Analysis

Uniform Manifold Approximation and Projection (UMAP) analysis was performed with the R package umap. K-means clustering analysis was performed with the in-built R function *kmeans*(). The number of clusters, k, was determined using the *fviz_nbclust*() (R package factoextra). Fold change over baseline was calculated as on-treatment sample value divided by baseline sample value. In case of values below lower limit of quantification, the value was set to the lower limit of quantification. Plasma cytokine levels were not normally distributed as tested by Shapiro-Wilk test and Skewness Kurtosis test for normality. The non-parametric two-sided Wilcoxon rank-sum test was used for comparison between two groups. The non-parametric Kruskal-Wallis rank-sum test was used for comparison between three or more groups. Time-to-event analysis was performed by the Kaplan-Meier method (R packages survival and survminer). Progression-free survival (PFS) was defined as time from treatment initiation to the date of first reported progression or death due to any course. Follow-up was defined as time from the date of treatment initiation to the last know date alive or the death date (for patients who had died during the study). Patients without disease progression or death were censored at the last follow-up date (October 2021). The two-side Log-rank test was used to assess differences in survival. Univariate and multivariable analyses were carried out by the Cox proportional hazard model. *p*-values below 0.05 were considered statistically significant. All analyses were carried out using R version 4.0.4 (R Studio, Boston, MA, USA).

## 3. Results

### 3.1. Patient Characteristics

A total of 77 patients diagnosed with unresectable stage III or IV melanoma at the Department of Oncology at Aarhus University Hospital or Aalborg University Hospital between August 2017 and June 2019 were enrolled in the study. All patients were treatment-naïve at the study start. Among the 77 patients, 29 (38%) patients received first-line treatment with anti-PD-1 inhibitor pembrolizumab (pembro), and 48 (62%) patients received first-line treatment with a combination of anti-PD-1 and anti-CTLA-4 inhibitors nivolumab plus ipilimumab (nivo/ipi) (Table 1). The two treatment groups were comparable for baseline characteristics, except for tumor PD-L1 expression (*p* < 0.0001) (Table 1). This discrepancy was caused primarily by the prescriptions which guide patients with tumor PD-L1 expression ≥ 1% for pembro, while patients having tumor PD-L1 expression < 1% were guided for nivo/ipi therapy. All patients had frozen plasma samples available at baseline, drawn before treatment initiation. One patient on pembro and five patients on nivo/ipi did not fulfill the requirement of having an on-treatment sample taken between one-to-three-month post-treatment initiation and were excluded from the analysis incorporating on-treatment samples. At the time of study evaluation, the median follow-up was 32 months (range 1.6–49.8 months). In the pembro group, 20/29 (69%) of the patients experienced disease progression, and during the follow-up time 13/29 (44.8%) died (Table 1). In the nivo/ipi group, 30/48 (62.5%) of the patients experienced disease progression, and during the follow-up time 24/48 (50%) died (Table 1).

### 3.2. Baseline Cytokine Profile Does Not Predict Response to Checkpoint Inhibitors

Cytokines are essential for the regulation and effector function of the immune system. Thus, they are often used as a read-out for various immune activation statuses in patients. As immune activation is required to benefit from checkpoint inhibitors, our first question was to address whether an inflammatory cytokine profile from a liquid biopsy taken before treatment initiation could predict treatment response as measured by time to progression. To test this, we designed a cytokine panel consisting of a total of 12 cytokines; 10 inflammatory cytokines known to reflect intrinsic immune activation status (IFNγ, IFNβ, IL6, IL8, IL10, CCL20, CXCL5, CXCL10, MCP1, and TNFα), as well as the soluble forms of the checkpoint molecules PD-1 and PD-L1, as these are the target for pembrolizumab and nivolumab, and the endogenous ligand for PD1, respectively.

Analysis of baseline cytokine levels across all samples demonstrated a dynamic expression range (Figure S1). However, stratifying patients into high and low expression did not associate with PFS for any cytokine (Figure S2).

**Figure 1:**
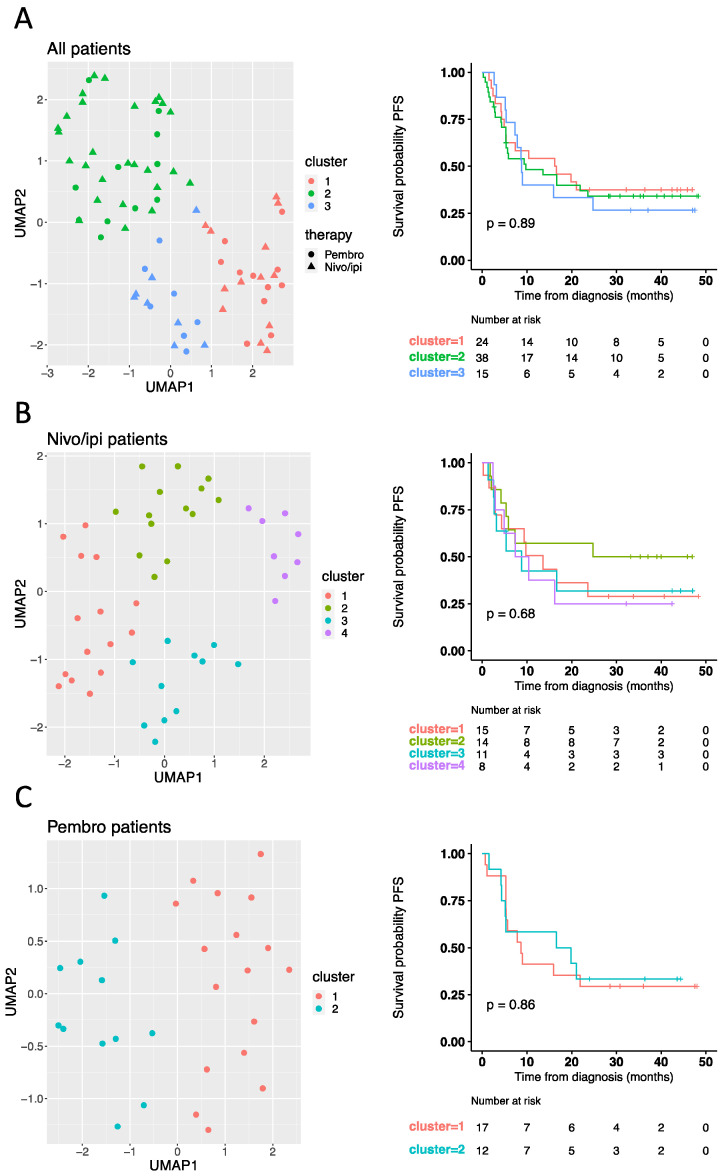
Baseline cytokine signature does not predict response to checkpoint inhibitors. Plasma levels of PD1, PD-L1, IFNγ, IFNβ, IL6, IL8, IL10, CCL20, CXCL5, CXCL10, MCP1, and TNFα were measured in baseline samples from all patients (*n* = 77). UMAP analysis based on all 12 cytokines was performed to visualize the distribution of all patients (**A**, **left panel**), nivo/ipi patients only (*n* = 48) (**B**, **left panel**), and pembro patients only (*n* = 29) (**C**, **left panel**), followed by k-means clustering analysis to divide patients into clusters. Clusters are represented by colors (**A**–**C**), whereas the shape of the points represents first-line therapy (**A**). (**A**–**C**, **right panel**) Kaplan-Meier curves showing the percentage of progression-free survival (PFS) by cytokine profile defined clusters. Statistical significance was tested with a log-rank test. Nivo/ipi, nivolumab plus ipilimumab; pembro, pembrolizumab.

We next hypothesized that multiple factors in combination would be more powerful to predict response to treatment as activation of the immune system is multifaceted and will support the regulation of many cytokines simultaneously. To perform an unbiased analysis of the cytokine panel and identify potential patterns within the data not directly visible by manual inspection, we decided to apply the dimensional reduction method UMAP. This approach allows high-dimensional data to be presented in a low-dimensional space while retaining the meaningful properties of the high-dimensional data [19]. First, we applied UMAP analysis to the cytokine profile obtained from the baseline samples from all 77 patients (Figure 1A, left panel). Using a k-means clustering approach, we identified and divided patients into three clusters, with the patients having the most similar cytokine profile clustering together. However, no association was observed between the identified clusters and PFS (log-rank *p* = 0.89) (Figure 1A, right panel). This led us to test the next hypothesis that a predictive cytokine profile could depend on the specific treatment regime. Thus, patients were divided into a nivo/ipi group (Figure 1B) and a pembro group (Figure 1C). For each group, we performed individual UMAP analyses based on the baseline cytokine profiles, followed by k-means clustering to identify clusters. However again, we did not observe an association between clusters and superior PFS for neither nivo/ipi patients (log-rank *p* = 0.68) (Figure 1B, right panel) nor pembro patients (log-rank *p* = 0.86) (Figure 1C, right panel). Altogether, these data suggest that a baseline inflammatory cytokine profile may not be useful as a predictive tool for how patients respond to checkpoint inhibitor therapy.

### 3.3. Checkpoint Inhibitor Treatment Induces an Inflammatory Cytokine Profile Distinct from Baseline and Healthy Donors

A generally accepted hypothesis is that any immune-activating effects induced by cancer treatment, which is the primary objective of checkpoint inhibitors, may accumulate over time. Thus, we speculated that evaluating cytokine levels during treatment rather than before treatment would be a better measure of treatment response.

**Figure 2:**
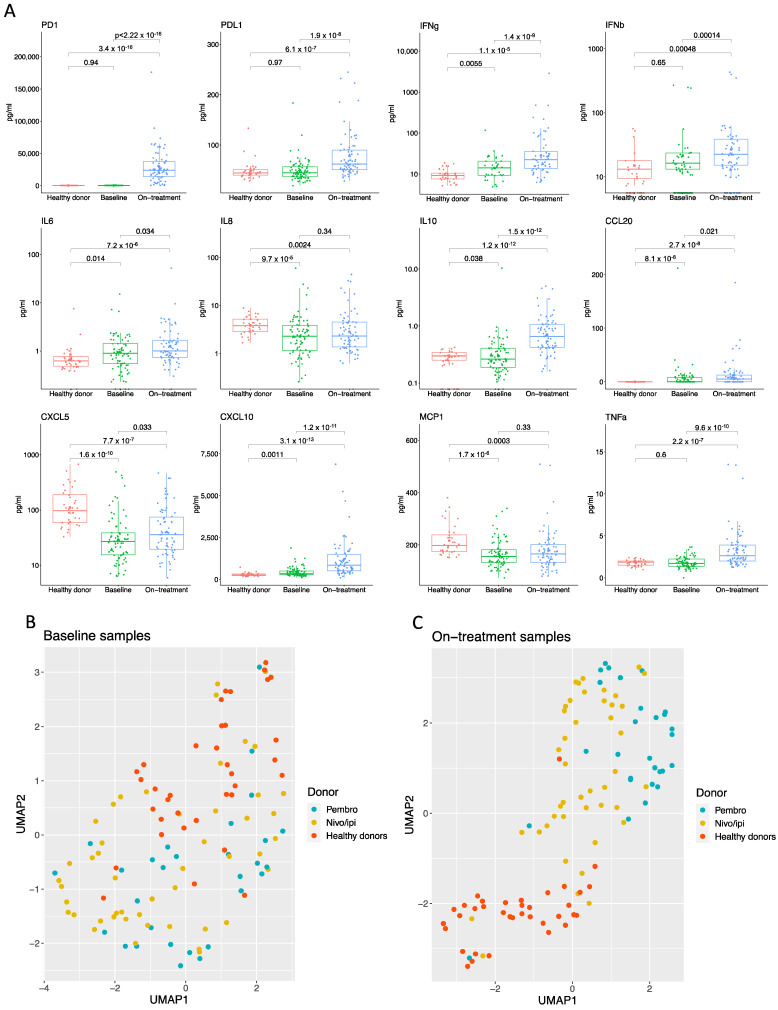
Checkpoint inhibitor therapy shapes distinct inflammatory cytokine profile. (**A**) Box plots showing levels of 12 cytokines measured in blood samples from healthy donors (*n* = 36), patient samples at baseline (*n* = 77), and patient samples taken on-treatment with either pembrolizumab or nivolumab and ipilimumab (*n* = 71). Statistical significance was tested with an unpaired Wilcoxon test. (**B**) UMAP analysis based on cytokine profile from healthy donors and baseline samples from pembro and nivo/ipi patients. (**C**) UMAP analysis based on cytokine profile from healthy donors (*n* = 36) and on-treatment samples from pembro (*n* = 28) and nivo/ipi patients (*n* = 43). Pembro, pembrolizumab; nivo/ipi, nivolumab plus ipilimumab.

To test this, we first compared the cytokine profile measured at baseline with the same cytokine profile measured in on-treatment samples. To control for general background levels of the measured cytokines, we introduced a group of age- and gender-matched healthy donors (*n* = 36). No difference was observed for PD1, PD-L1, IFNβ, and TNFα when comparing the individual cytokine levels between healthy donors and patients’ baseline samples (Figure 2A). In contrast, the baseline levels of IL8, IL10, CXCL5, and MCP1 were significantly lower in patients compared with healthy donors (Figure 2A). Finally, the baseline levels of IFNγ, IL6, IL10, CCL20, and CXCL10 were found significantly higher in patients than in healthy donors (Figure 2A).

Interestingly, when investigating the signals from the patients’ on-treatment samples, we observed that for all cytokines, except IL8, CXCL5, and MCP1, the levels were significantly higher compared to patients’ baseline samples and healthy donors (Figure 2A). This suggests that checkpoint inhibitor treatment induces a systemic and measurable cytokine induction early after treatment initiation.

We next performed a UMAP analysis based on the healthy donors and the baseline samples from all patients (Figure 2B). In agreement with the observations in Figure 2A, the UMAP analysis demonstrated that healthy donors and patients clustered together, suggesting that their cytokine profiles were similar (Figure 2B). Next, we performed a UMAP analysis based on the cytokine profile from healthy donors and patients’ on-treatment samples (Figure 2C). Interestingly, we observed that patients and healthy donors were now divided into two distinct clusters, indicating an overall difference in the cytokine profile between healthy donors and patients (Figure 2C). Moreover, the data also showed a tendency of patients to cluster depending on the therapy regime (Figure 2C turquoise and yellow). A separate UMAP analysis excluding the healthy donors, followed by unsupervised k-means clustering, confirmed the treatment-dependent clustering of patients with one cluster (green) containing almost all pembro-treated patients and another cluster (yellow) containing the majority of patients treated with nivo/ipi (Figure S3A,B). In summary, these analyses demonstrate that treatment with checkpoint inhibitors induced significant changes in the measured cytokines compared to baseline levels, and these changes are specific to the treatment used.

### 3.4. On-Treatment Cytokine Profile Predicts Response to Checkpoint Inhibitors

**Figure 3:**
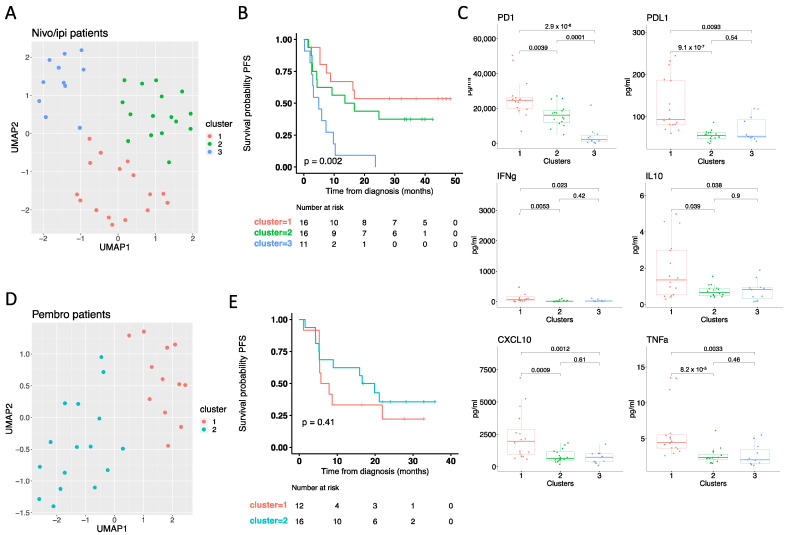
On-treatment cytokine profile predicts response to nivolumab and ipilimumab. (**A**) UMAP analysis of cytokine profiles in on-treatment samples from nivo/ipi patients (*n* = 43). Colors indicate clusters identified by k-means clustering. (**B**) Kaplan-Meier curve showing the percentage of progression-free survival (PFS) of nivo/ipi patients by clusters defined in panel (**A**). Statistical significance was tested with a log-rank test. (**C**) Box plots showing plasma levels of PD1, PD-L1, IFNγ, IL10, CXCL10, and TNFα measured in on-treatment samples from nivo/ipi patients, according to clusters defined in panel (**A**). Statistical significance was tested with an unpaired Wilcoxon test. (**D**) UMAP analysis of cytokine profiles in on-treatment samples from pembro patients (*n* = 28). Colors indicate clusters identified by k-means clustering. (**E**) Kaplan-Meier curve showing the percentage of PFS of pembro patients by clusters defined in panel (**D**). Statistical significance was tested with a log-rank test. Nivo/ipi, nivolumab plus ipilimumab; pembro, pembrolizumab.

As our results indicate that the cytokine profile measured in on-treatment samples is significantly affected by the treatment and that the two treatment modalities induce distinct cytokine profiles, we next speculated that such changes could be predictive of the response to therapy. To evaluate this, we performed an individual UMAP analysis for each treatment group based on the on-treatment samples (Figure 3A,D). A total of 43 patients treated with first-line nivo/ipi had an available on-treatment sample from which a UMAP analysis was performed. Using k-means clustering, we identified three different clusters of patients (Figure 3A). We then tested if any of the clusters were associated with superior PFS and found that patients in clusters 1 and 2 had significantly longer PFS than patients in cluster 3 (log-rank *p* = 0.002) (Figure 3B). Given that patients in clusters 1 and 2 had superior PFS, we next deconvoluted the UMAP output to identify which cytokines were associated with the clusters. This identified PD1, PD-L1, IFNγ, IL10, CXCL10, and TNFα to be significantly higher in patients from cluster 1 compared to patients from cluster 3, whereas PD-1 was the only cytokine significantly different between clusters 2 and 3 (Figure 3C). For the remaining cytokines (CCL20, CXCL5, IFNβ, IL6, IL8, and MCP1), patients in clusters 1 and 2 showed comparable levels to patients in cluster 3 (IL6, CCL20, and CXCL5) (Figure S4A).

The same analysis for patients treated with first-line pembro, identified two clusters of patients (Figure 3D), but none of them were associated with superior PFS (log-rank *p* = 0.41) (Figure 3E and Figure S4B,C). Taken together, this demonstrates that high levels of PD1, PD-L1, IFNγ, IL10, CXCL10, and TNFα are associated with superior PFS in our cohort of patients treated with nivo/ipi, but we were unable to identify a cytokine profile correlating to superior PFS for patients treated with pembro. This could potentially be due to the limited sample size in our cohort.

**Figure 4:**
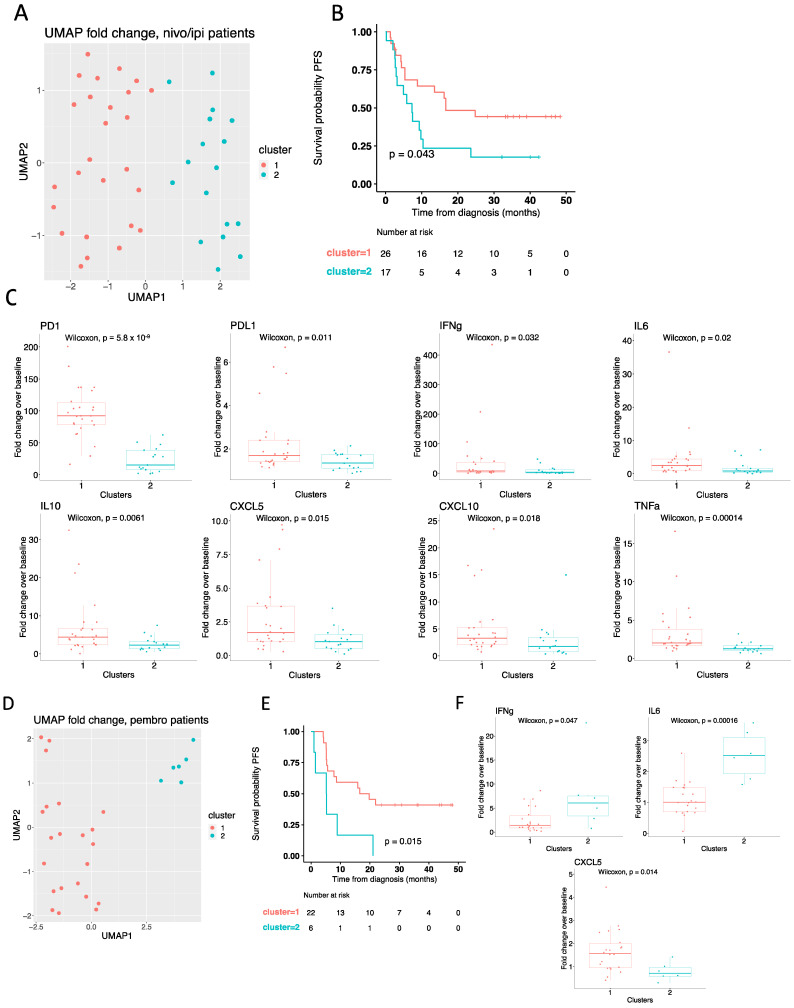
Treatment-induced cytokine changes predict response to checkpoint inhibitors. (**A**) UMAP analysis of nivo/ipi patients (*n* = 43) based on the fold change over baseline for all 12 cytokines. Colors indicate clusters defined by k-means clustering. (**B**) Kaplan-Meier curve showing the percentage of progression-free survival (PFS) for nivo/ipi patients by clusters defined in panel (**A**). Log-rank test was used to test for statistical significance. (**C**) Box plots showing the fold change over baseline for PD1, PD-L1, IFNγ, IL6, IL10, CXCL5, CXCL10, and TNFα in nivo/ipi patients by clusters defined in panel A. Statistical significance was tested with an unpaired Wilcoxon test. (**D**) UMAP analysis of pembro patients (*n* = 28) based on the fold change over baseline for all 12 cytokines. Colors indicate clusters defined by k-means clustering. (**E**) Kaplan-Meier curve showing the percentage of PFS for pembro patients by clusters defined in panel (**D**). Log-rank test was used to test for statistical significance. (**F**) Box plots showing the fold change over baseline for IFNγ, CXCL5, and IL6 in pembro patients by clusters defined in panel (**D**). Statistical significance was tested with an unpaired Wilcoxon test. Nivo/ipi, nivolumab plus ipilimumab.

Developing a prediction algorithm based on raw cytokine values will be problematic as such data are dependent on assays and machinery robustness. To overcome this, we calculated cytokine values as a fold change from on-treatment over baseline levels and used this in our UMAP algorithm. For the 43 nivo/ipi-treated patients, the UMAP analysis, followed by k-means clustering analysis, identified two clusters (Figure 4A), with patients in cluster 1 having significantly longer PFS compared with patients from cluster 2 (log-rank *p* = 0.043) (Figure 4B). Deconvolution of the clusters revealed that cluster 1 was associated with significantly higher fold changes of PD1, PD-L1, IFNγ, IL6, IL10, CXCL5, CXCL10, and TNFα (Figure 4C). In contrast, IFNβ, IL8, CCL20, and MCP1 were not significantly different between the two clusters (Figure S5A).

A similar analysis was performed for patients treated with first-line pembro, resulting in the definition of two distinct clusters (Figure 4D). Subsequent survival analysis demonstrated superior PFS for patients in cluster 1 compared with patients in cluster 2 (log-rank *p* = 0.015) (Figure 4E). Finally, deconvolution revealed that patients in cluster 1 were characterized by significantly higher fold changes of CXCL5 and significantly lower fold changes of IFNγ and IL6 compared with patients in cluster 2 (Figure 4F). No difference in fold changes was observed for the remaining cytokines (Figure S5B).

### 3.5. High PD-1 Increment Predicts Response to Nivolumab plus Ipilimumab

Based on our data, demonstrating that high numerical levels and high increments of PD1, PD-L1, IFNγ, IL6, IL10, CXCL5, CXCL10, and TNFα in on-treatment samples were characteristic of clusters with superior PFS in the nivo/ipi group (Figure 3C and Figure 4C), we wanted to evaluate the individual predictive value of these three cytokines. To test this, we performed a univariate and multivariable cox regression analysis on nivo/ipi-treated patients with available on-treatment samples. The included parameters were age (continuous variable), gender, lactate dehydrogenase (LDH) levels (normal vs. elevated), performance status (0 vs. 1 or 2), and PD1, PD-L1, IFNγ, IL6, IL10, CXCL5, CXCL10, and TNFα each dichotomized by median fold change into two groups. The univariate analysis revealed that high fold change of PD-1 (HR = 0.35; 95% Confidence interval (CI) 0.16–0.77; *p* = 0.0086), but not PD-L1, IFNγ, IL6, IL10, CXCL5, CXCL10, and TNFα, was a predictor for longer PFS (Table 2). This was confirmed in a multivariate analysis demonstrating that the high fold change of PD-1 (HR = 0.13; 95% CI 0.034–0.49; *p* = 0.0026) was a predictor for PFS (Table 2).

Next, we wanted to test the individual predictive value of IFNγ, CXCL5, and IL6, which were significantly different between the two clusters found in the pembro group (Figure 4C). As for the nivo/ipi group, we performed a univariate and multivariable cox regression analysis on the pembro group, including the parameters age, gender, LDH, performance status, and IFNγ, CXCL5, and IL6 each dichotomized by median fold change into two groups (Table 3). However, none of the three cytokines identified from the deconvolution of UMAP clusters were significantly correlated to PFS (Table 3).

In summary, this demonstrated that the ratio values of soluble PD1 in plasma from baseline to early during treatment could be used as a strong predictor of PFS in patients treated with nivo/ipi. In contrast, none of the three cytokines (IFNγ, CXCL5, and IL6) identified in the pembro group were significantly associated with PFS.

## 4. Discussion

Here, we evaluated the association between PFS and the concentration and kinetics of 12 plasma cytokines measured before and early during treatment with pembrolizumab or nivolumab plus ipilimumab in advanced-stage melanoma patients. The cytokines were selected based on their association with immune function in the context of cancer and checkpoint inhibitor therapy.

Importantly, we did not observe any correlation between baseline cytokine levels and response to checkpoint inhibitors. In contrast, we did observe a treatment-specific change in the cytokine profile upon treatment initiation supporting the hypothesis that immunological activation and changes in tumor tissues can be monitored by liquid biopsies. Using an unbiased approach focusing on UMAP and cluster analysis, we demonstrated that high increments of soluble PD-1, PD-L1, IFNγ, IL10, CXCL10, and TNFα measured during treatment were characteristic for patient clusters associated with superior survival in the nivo/ipi group. Cox regression analysis including these cytokines revealed that PD-1 was a strong individual predictor for PFS. A similar analysis of the pembro group, demonstrated that the patient-cluster with superior PFS was characterized by low increments of IFNγ and IL6 and high increments of CXCL5. However, none of these cytokines individually predicted PFS.

Much research in the field of checkpoint inhibitor therapy for cancer is focused on identifying biomarkers that can predict response. Currently, pre-treatment tumor PD-L1 expression is the only clinically used biomarker for checkpoint inhibitors in melanoma, but it is associated with low specificity [3,20]. During recent years, much focus has been on identifying biomarkers in liquid biopsies, such as blood samples, as these are easy and minimal invasive to sample. Several papers have evaluated the correlation between baseline blood levels of cytokines or soluble PD-1 and PD-L1, and response to checkpoint inhibitors in melanoma, resulting in the proposal of several cytokines as predictive biomarkers [12,13,16,17,18,21,22]. In the present study, we did not identify any correlation between baseline cytokine levels and PFS, independent of the first-line therapy. This discrepancy can perhaps be explained by differences in sampling method, such as plasma versus serum, which is known to affect cytokine levels [23,24], composition of the cohorts, treatment modalities, and pre-treatment status of included patients. Importantly, our cohort only includes treatment-naïve patients, whereas other cohorts include a considerable part of pre-treated patients [15,17,18,21,22]. There is plenty of evidence demonstrating that cytokine levels are highly affected by prior systemic treatment, with PD-1 being particularly increased upon systemic treatment [15,17,22,25]. Thus, prior systemic treatment may influence the analysis of cytokines as predictive biomarkers and can explain the missing correlation between baseline cytokine levels and PFS observed in this cohort, compared with previous studies. Comparing patients’ baseline samples with a healthy control group using UMAP analysis, we found that the overall cytokine profile was comparable, suggesting that melanoma patients do not show any specific immune active cytokine signature at baseline. Hence, baseline cytokine profiles in treatment-naïve patients are most likely not an optimal biomarker for response to treatment, merely because the treatment effect is not yet reflected in the cytokine levels of the patients.

Given that checkpoint inhibitors work by reinvigorating a previous active anti-tumor immune response, we hypothesized that treatment-induced cytokine changes could reflect treatment efficacy. Interestingly, we found that the patient-clusters in the nivo/ipi group with superior PFS generally were characterized by higher cytokine levels, suggesting a more robust immune activation in these patients. Furthermore, we found a high fold change of PD-1 early during treatment to be a strong individual predictor for PFS in patients treated with nivo/ipi. This finding agrees with previous reports, showing that increased or steady levels of PD-1 upon treatment initiation (with erlotinib or nivolumab) are associated with superior PFS in non-small cell lung cancer (NSCLC) [26,27]. Although the source is unknown, soluble PD-1 in blood has been linked to increased CD8 T cell activity and anti-tumor immunity [28,29,30,31]. Together, this supports the concept that increasing levels of cytokines related to anti-tumor immunity can be a specific measure of treatment response. Furthermore, this highlights the importance of evaluating on-treatment samples, as these have been influenced by the treatment effect, and therefore may be valuable for monitoring treatment response and potentially disease progression.

We did not find high increments of PD-1 to be associated with superior PFS in the pembro group. This is consistent with two recent studies demonstrating elevated PD-1 levels after pembrolizumab treatment but no correlation to PFS [17,32]. Instead, we found that the patient cluster with superior PFS was characterized by low fold changes of IFNγ and IL6 and high fold change of CXCL5. This discrepancy between the two treatment modalities may be explained by the addition of the anti-CTLA-4 inhibitor to the combination therapy resulting in distinct genetic and immunological changes as described previously [33]. Such treatment-specific biomarkers could potentially guide patients toward the best possible therapy. Given that the three identified cytokines were not individually associated with PFS, it suggests that combining multiple cytokines into one model may be a better predictor for PFS for pembrolizumab, but this needs further validation.

Although the presented study provides new data on specific cytokines as predictive biomarkers and the importance of evaluating on-treatment samples, we are aware of certain limitations in the study. The study was restricted in sample size, making it impossible to split the cohort into a discovery and validation cohort. Hence, our findings will need validation in treatment-naïve patient cohorts with available plasma samples at baseline and early during treatment. This has, so far, not been possible to identify. However, it is worth noting that the association between elevated soluble PD-1 and PFS has been demonstrated before, albeit in NSCLC [26,27]. We also acknowledge that we did not perform a large-scale cytokine analysis but focused on a selected panel of merely 12 cytokines. Therefore, we cannot exclude that other systemic inflammatory cytokines may improve specificity and sensitivity for predicting treatment response. Finally, owing to the treatment guideline valid at the time the study was conducted, the majority of patients in the nivo/ipi treatment group had PD-L1 negative tumors. Thus, we cannot conclude that increases in PD-1 are associated with superior PFS in patients having PD-L1 positive tumors and being treated with nivo/ipi. This will require further investigations.

## 5. Conclusions

In conclusion, in this cohort of melanoma patients entitled to checkpoint inhibitor therapy, we demonstrate that a high fold change of PD-1 is a strong individual predictor for PFS in the nivo/ipi group. In contrast, a model including fold changes of IFNγ, IL6, and CXCL5 is associated with superior PFS in the pembro group. It is possible that these biomarkers have clinical implications and can be used to enable better guidance of treatment modality, thereby increasing the chances of successful treatment and preventing the exposure of patients to unnecessary serious adverse event.

## Figures and Tables

**Table 1:** Patient characteristics. Patient characteristics, besides progression and alive, refers to the status at baseline. A Chi-square test was used for statistical comparison between groups. AJCC, American Joint Committee on Cancer; Programmed death-ligand 1; PD-1, Programmed cell death protein 1; CTLA-4, cytotoxic T-lymphocyte-associated protein 4; LDH, Lactate dehydrogenase; N/A, not available.

Parameter	Pembrolizumab (anti-PD1)	Nivolumab/Ipilimumab (anti-PD1/CTLA-4)	*p*
Total	29	48	
Age, year mean (range)	68 (31–89)	60 (30–85)	
Sex, n (%) - Male - Female	18 (62) 11 (38)	29 (60.4) 19 (39.6)	0.89
AJCC stage, n (%) - Stage III - Stage IV	3 (10.3) 26 (89.7)	5 (10.4) 43 (89.6)	0.99
BRAF status, n (%) - Wild type - Mutated	16 (55.2) 13 (44.8)	26 (54.2) 22 (45.8)	0.93
Tumor PD-L1 expression, n (%) - < 1% - ≥ 1% - N/A	4 (13.8) 22 (75.9) 3 (10.3)	43 (89.6) 2 (4.2) 3 (6.2)	<0.0001
Serum LDH, n (%) - Normal - Elevated - N/A	21 (72.4) 6 (20.7) 2 (6.9)	32 (66.7) 14 (29.2) 2 (4.1)	0.65
Progression, n (%) - No - Yes	9 (31) 20 (69)	18 (37.5) 30 (62.5)	0.56
Alive, n (%) - No - Yes	13 (44.8) 16 (55.2)	24 (50) 24 (50)	0.81

**Table 2:** Cox regression analysis for the nivo/ipi group (*n* = 43).

Parameters	PFS	
	Hazard Ratio (95% Confidence Interval)	*p*
**Univariate Cox regression analysis**		
Age	1 (0.97–1.03)	0.92
Gender (Female vs male)	1.13 (0.54–2.48)	0.75
LDH (normal vs elevated)	0.56 (0.24–1.32)	0.18
Performance status (ECOG) (0 vs 1 or 2)	2.19 (1.06–4.53)	0.034
PD-1 fold change (above vs below median)	0.35 (0.16–0.77)	0.0086
CXCL10 fold change (above vs below median)	1.15 (0.55–2.41)	0.71
TNFα fold change (above vs below median)	1.09 (0.52–2.29)	0.82
PD-L1 fold change (above vs below median)	1.27 (0.60–2.66)	0.53
IFNγ fold change (above vs below median)	0.98 (0.47–2.07)	0.97
IL10 fold change (above vs below median)	0.94 (0.45–1.98)	0.87
**Multivariable Cox regression analysis**		
Age	0.98 (0.95–1.026)	0.49
Gender (Female vs male)	1.042 (0.43–2.5)	0.93
LDH (normal vs elevated)	0.16 (0.041–0.64)	0.0096
Performance status (ECOG) (0 vs 1 or 2)	2.41 (0.81–7.19)	0.12
PD-1 fold change (above vs below median)	0.13 (0.0034–0.49)	0.0026
CXCL10 fold change (above vs below median)	2.43 (0.68–8.71)	0.17
TNFα fold change (above vs below median)	0.81 (0.18–3.69)	0.79
PD-L1 fold change (above vs below median)	0.46 (0.14–1.5)	0.20
IFNγ fold change (above vs below median)	1.42 (0.53–3.82)	0.48
IL10 fold change (above vs below median)	2.095 (0.51–8.53)	0.30

**Table 3:** Cox regression analysis for the pembro group (*n* = 28).

Parameters	PFS	
	Hazard Ratio (95% Confidence Interval)	*p*
**Univariate Cox regression analysis**		
Age	1 (0.97–1.28)	0.89
Gender (Female vs male)	1.84 (0.71–4.80)	0.21
LDH (normal vs elevated)	2.17 (0.77–6.079)	0.14
Performance status (ECOG) (0 vs 1 or 2)	1.72 (0.69–4.31)	0.24
IFNγ fold change (above vs below median)	1.74 (0.69–4.34)	0.24
CXCL5 fold change (above vs below median)	0.64 (0.26–1.6)	0.34
IL6 fold change (above vs below median)	0.64 (0.26–1.6)	0.34
**Multivariable Cox regression analysis**		
Age	0.99 (0.95–1.024)	0.47
Gender (Female vs male)	2.21 (0.67–7.24)	0.19
LDH (normal vs elevated)	1.74 (0.50–6.052)	0.38
Performance status (ECOG) (0 vs 1 or 2)	0.91 (0.3–2.81)	0.87
IFNγ fold change (above vs below median)	1.91 (0.45–8.032)	0.38
CXCL5 fold change (above vs below median)	0.8 (0.23–2.75)	0.72
IL6 fold change (above vs below median)	0.66 (0.21–2.067)	0.48

## Data Availability

All data relevant to the study are included in the article or uploaded as online supplemental information.

## Supplementary Materials

The following supporting information can be downloaded at: https://www.mdpi.com/article/10.3390/cancers14143342/s1, Figure S1: Baseline cytokine expression; Figure S2: Baseline cytokine expression correlation to PFS; Figure S3: Treatment modalities shape distinct cytokine profiles in on-treatment samples; Figure S4: Deconvolution of UMAP clusters based on on-treatment samples; Figure S5: Deconvolution of UMAP clusters based on cytokine fold change over baseline.

## Abbreviations

OS	Overall survival
PFS	Progression-free survival
HR	Hazard ratio
CI	Confidence interval
Pembro	Pembrolizumab
Nivo/ipi	Nivolumab plus ipilimumab
MM	Malignant melanoma
ECOG	Eastern Cooperative Oncology Group
U/L	Units/liter
LHD	Lactate dehydrogenase
CRP	C-reactive protein
PD-L1	Programmed death ligand 1
EDTA	Ethylenediaminetetraacetic acid
IFN	Interferon
IL	Interleukin
CXCL10	C-X-C motif chemokine ligand 10
CCL20	Chemokine (C-C-motif) ligand 20
TNF	Tumor Necrosis factor
MCP	Monocyte Chemoattractant Protein 1
PD-1	Programmed cell death protein 1
CTLA-4	Cytotoxic T-lymphocyte-associated protein 4
UMAP	Uniform Manifold Approximation and Projection
CD8	Cluster of differentiation 8
TGF-β	Transforming growth factor β
PET/CT	Position emission tomography/computed tomography
MRI	Magnetic resonance imaging
NSCLC	Non-small cell lung cancer
