# Supplementary material for: Increased Soluble PD-1 Predicts Response to Nivolumab plus Ipilimumab in Melanoma
Source: Cancers (Basel). 2022 Jul 9;14(14):3342. doi: 10.3390/cancers14143342 (PMC9322974; doi:10.3390/cancers14143342)
Supplement: Supplementary file 1 [file cancers-14-03342-s001.zip › cancers-1777428 supplementary.pdf]

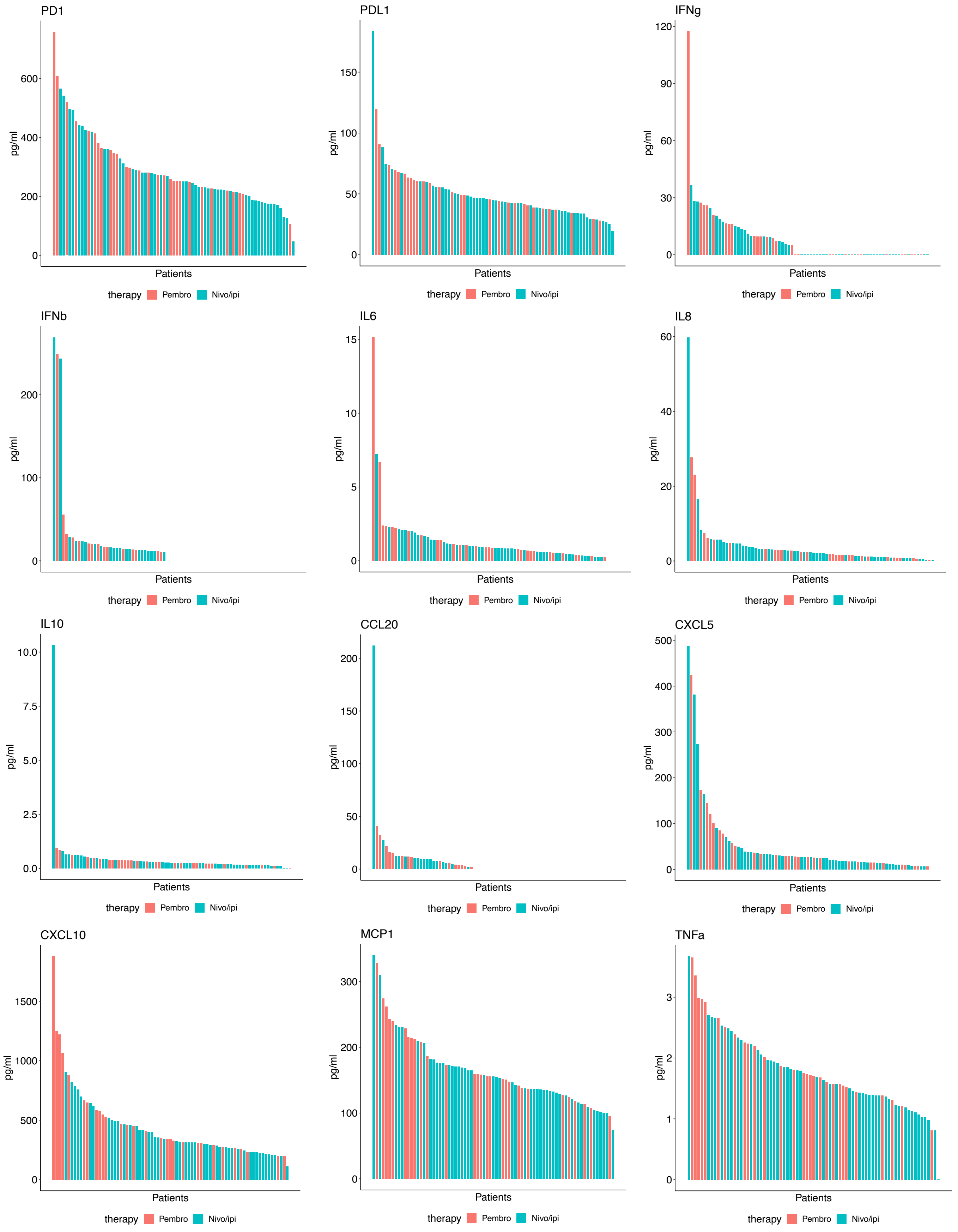

Figure S1. Baseline cytokine expression: Waterfall plots showing baseline cytokine expression for each individual cytokine. Colors indicate first-line therapy. (n = 77). Pembro, pembrolizumab; nivo/ipi, nivolumab plus ipilimumab.

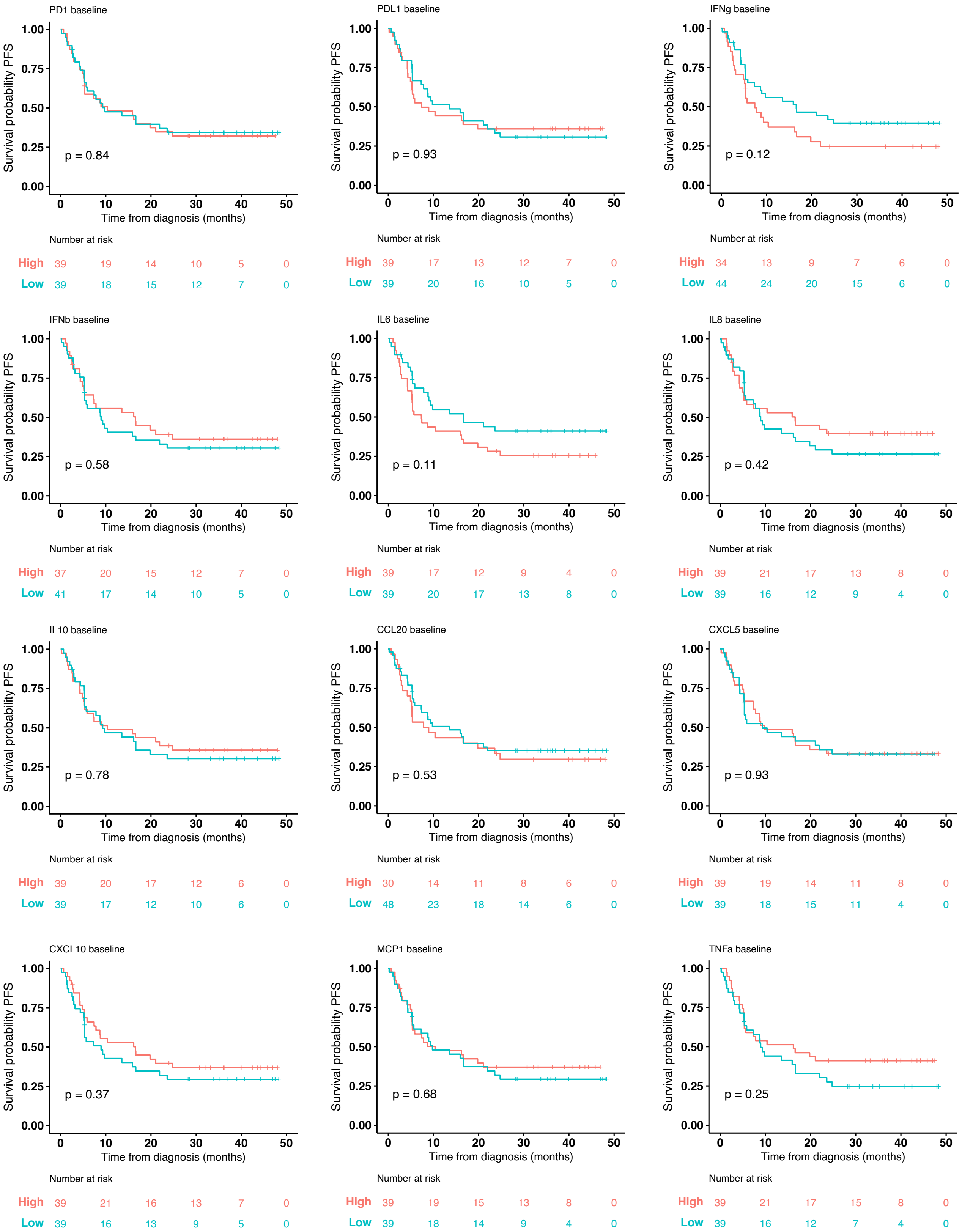

Figure S2. Baseline cytokine expression correlation to PFS: For each cytokine, the patients (n = 77) were dichotomized into a low and high group by the median baseline cytokine expression levels and correlated to PFS using the Kaplan-Meier method. Statistical significance was tested with a log-rank test.

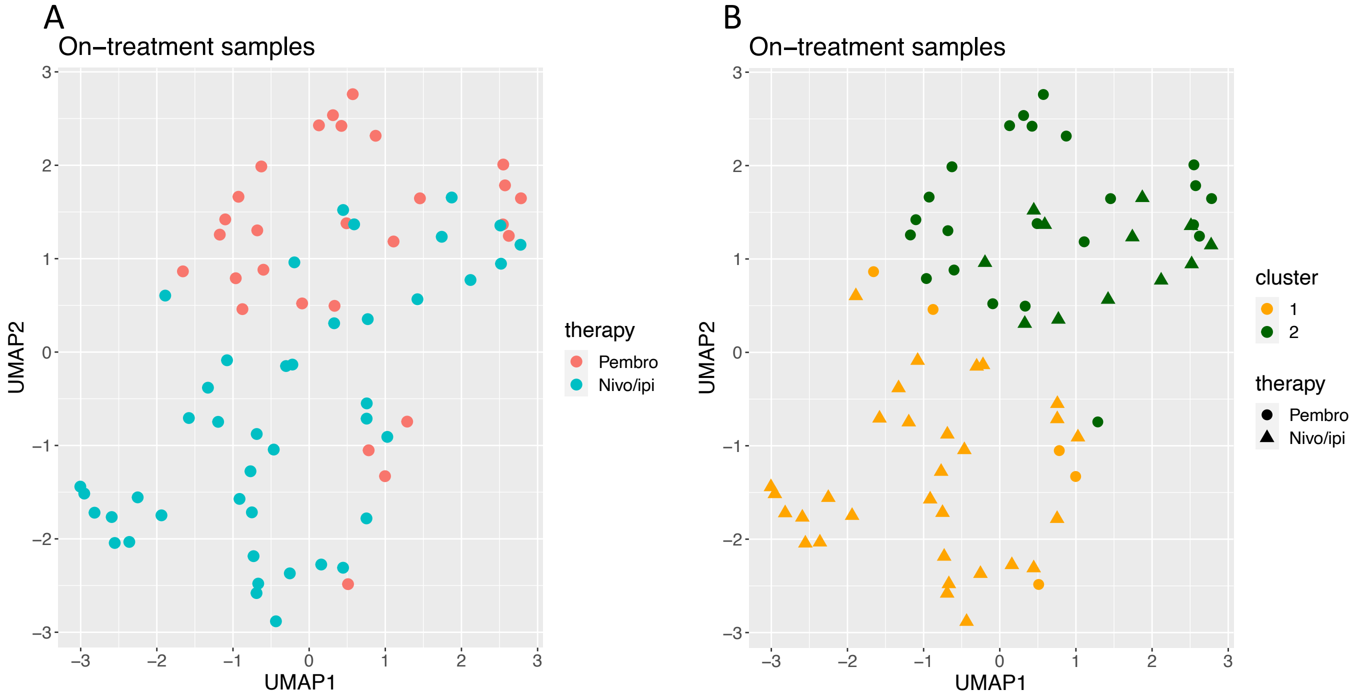

Figure S3. Treatment modalities shape distinct cytokine profiles in on-treatment samples. A+B) UMAP plots based on the cytokine profile measured in on-treatment samples from patients treated with either pembrolizumab or nivolumab plus ipilimumab (n = 71). A) Colors indicate first-line treatment. A separation of patients according to first-line treatment is seen across the X2 axis. B) Colors indicate clusters defined by k-means clustering analysis performed on the UMAP plot from panel A. Pembro, pembrolizumab; nivo/ipi, nivolumab plus ipilimumab.

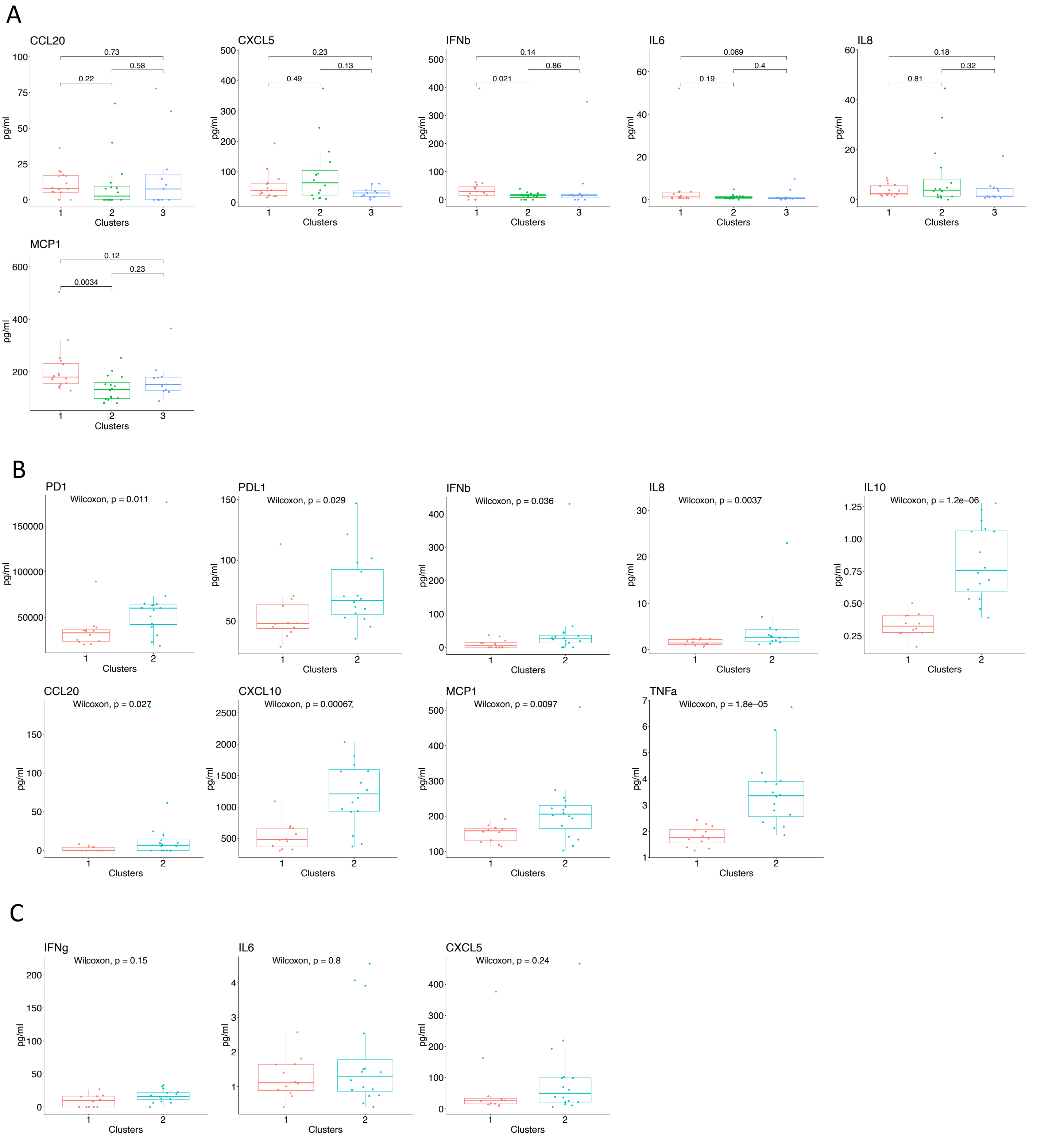

Figure S4. Deconvolution of UMAP clusters based on on-treatment samples. A) Box plots show-ing deconvolution of UMAP cluster based on on-treatment samples from nivolumab plus ipili-mumab patients (n = 43). Each cytokine was plotted against patient-clusters defined in figure 3A. Statistical significance was tested with an unpaired Wilcoxon test. B + C) Box plots showing decon-volution of UMAP cluster based on on-treatment samples from pembrolizumab patients (n = 28). Each cytokine was plotted against patient-clusters defined in figure 3D. Statistical significance was tested with an unpaired Wilcoxon test. B) Box plots showing cytokines significantly higher in cluster 2 compared with cluster 1. C) Box plots showing cytokines with no significant difference between clusters.

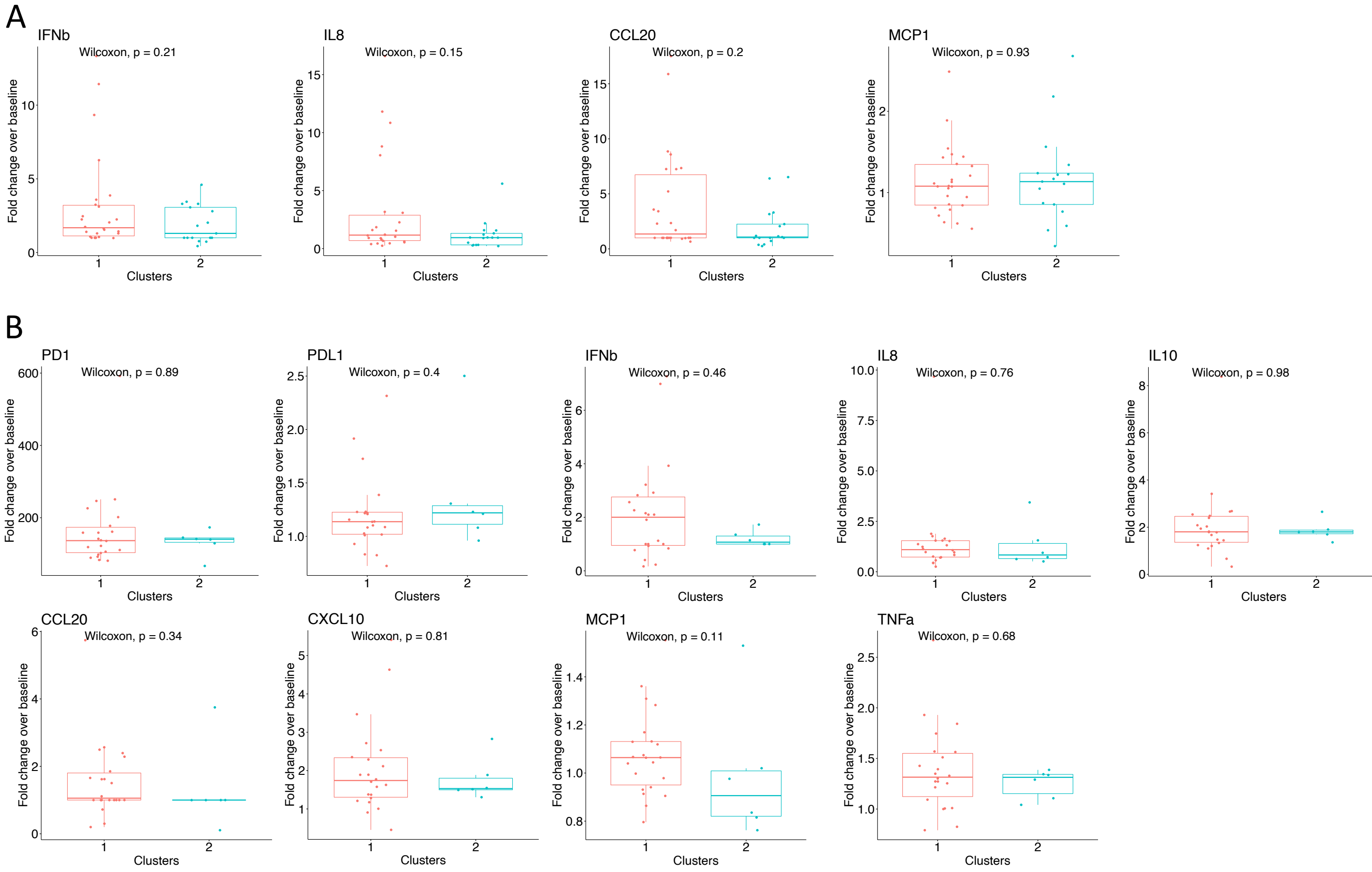

Figure S5. Deconvolution of UMAP clusters based on cytokine fold change over baseline. A) Box plots showing deconvolution of UMAP cluster based on cytokine fold change in nivolumab plus ipilimumab patients (n = 43). Each cytokine was plotted against patient-clusters defined in figure 4A. Statistical significance was tested with an unpaired Wilcoxon test. B) Box plots showing decon-volution of UMAP cluster based on cytokine fold change in pembrolizumab patients (n = 28). Each cytokine was plotted against patient-clusters defined in figure 4D. Statistical significance was tested with an unpaired Wilcoxon test.
